# The importance of particle-support interaction on particle size determination by gas chemisorption

**DOI:** 10.1007/s11051-016-3385-2

**Published:** 2016-03-23

**Authors:** L. Torrente-Murciano

**Affiliations:** Department of Chemical Engineering and Biotechnology, University of Cambridge, Cambridge, CB2 3RA UK

**Keywords:** Metal nanoparticle, Metal-support interaction, Particle shape, chemisorption, Particle size, Modelling and simulations

## Abstract

**Abstract:**

The interaction of the metal-support and particle shape has a key role on the determination of the particle size by gas chemisorption. This paper demonstrates mathematically that, assuming metal particles with hemispherical shapes (a common assumption in this type of characterisation) can provide misleading results of up to one order of magnitude. Thus, the metal particle sizes are underestimated when the metal strongly interacts with the support and overestimated when there is a weak metal-support interaction. Additionally, we also demonstrate that although the assumption of spherical shapes always underestimates the size of particles, this error is considerably lower with regular geometries than that associated to the effect of the metal-support interaction due to their effect on the particle shape. Herein, it is demonstrated the importance of introducing the particle-support interaction factor in the chemisorption particle size determination.

**Graphical Abstract:**

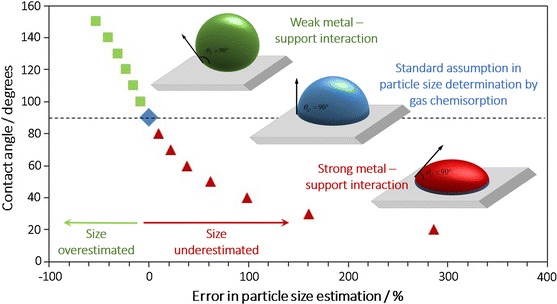

## Introduction

The discovery, characterisation and understanding of the unique properties of materials with dimensions between 10^−9^ and 10^−10^ m (nano- and sub-nano scale) is currently one of the most active research areas due to their potential application in a wide range of fields from catalysis, sensors, biological labelling, photonics, plasmonics, data storage (Pankhurst et al. 2003; Panyam and Labhasetwar 2003), etc. Specifically, metal clusters and particles present unique chemical and physical properties which are strongly dependent on their size (Kelly et al. 2003; Daniel and Astruc 2004). Focusing on catalysis, the strong relationship between the size of the metal particle and its activity and selectivity has been demonstrated for multiple systems (Panigrahi et al. 2007; Wang et al. 2008). Furthermore, for a given reaction and metal, an optimum particle size range exists outside which its reactivity is considerably lower or even negligible (Schmid 2008). In addition to this, computational chemists, aided by advanced characterisation techniques, have also revealed the key relationship between catalytic activity and shape of the metal nanoparticles due to the selective exposure of different cluster facets and their effect on the adsorption strength of reactants. Indeed, several authors support the fact that the high activity of the metal nanoparticles is directly related to the high concentration of uncoordinated atoms in nano-sized particles (Lemire et al. 2004), further promoted by specific 3D shapes (Molina et al. 2011; Yasumatsu and Fukui 2015). For example, it is known that the optical response from metal nanoparticles and nanostructures is dominated by surface plasmon generation and is critically dependent on the local structure and geometry such as a range of polyicosahedral shapes or ellipsoids (Rossi et al. 2004; Bosman et al. 2007). As a consequence, control of the metal particle size and shape in addition to accurate characterisation are crucial for further development in the field.

High-resolution transmission electron microscopy (TEM) provides qualitative and semi-quantitative information about the size distribution and shape of the metal particles as well as their dispersion across the support. However, the contrast in electron microscopy is dependent on the ratio of the atomic numbers of the metal and the support, with small particles having a lower contrast than big ones. Particles with diameters below 1–1.5 nm are considerably more difficult to detect, limiting the accurate quantification of the particle size distribution. In the last decade, the development of aberration corrected scanning transmission electron microscopes and work carried out using high-angle annular dark field detectors have considerably improved the quantification of particle sizes in the sub-nanometre scale (Herzing et al. 2008; Hetherington et al. 2008). However, the high cost of this type of characterisation limits the use of these facilities for routine particle size determination. Gas chemisorption, normally using H_2_ and CO as probe molecules, is widely used either in combination with TEM to provide quantification of the particle size distribution or by itself to calculate the metallic surface area accessible to the probe molecule. The technique consists of the measurement of the number of probe molecules adsorbed on the metallic surface of a material. By knowing the stoichiometry factor of the number of adsorbed probe molecules per metallic surface atom, the metallic surface area, average particle size and metal dispersion can be calculated. It is widely accepted that one of the main limitations of gas chemisorption as a particle size determination technique is the accurate determination of the mentioned stoichiometry factor which strongly depends on the arrangement of the surface atoms. In fact, the probe molecule can form linear, twofold or threefold bridged adducts, ranging its value from 0.5 to 2 for a given metal (Stara et al. 1996; Henry 1998; Ozensoy and Goodman 2004). This paper further demonstrates that the interaction of the metal and its support (i.e. the contact angle between both) can have a more important effect on the resulting average particle size determination due to the conventional assumption of the semi-spherical shape of the particle than the stoichiometric factor. Additionally, it also demonstrates the error associated to the assumption of spherical shapes when different 3D metal structures are present in the sample. New calculations are proposed to consider the morphology of the metal particle and the contact angle with the support for accurate size determination using easily accessible chemisorption characterisation.

## Results and discussion

Let’s consider a case study where metal nanoparticles are supported on an inner support where pulse chemisorption, using carbon monoxide as probe molecule, is used as a characterisation technique to estimate the average particle size. In the standard procedure of this technique, known amounts of CO are pulsed into the sample under a flow of inert gas. The amount of CO adsorbed in each pulse is quantified by analysing the outlet gas concentration using a thermal conductivity detector (TCD) or a similar detector. The pulses are repeated until the sample is fully saturated with the probe molecule. The total amount of CO adsorbed per mass of sample is denoted as *V*_ads_ (cm^3^ g^−1^).

The metallic surface area (SA_metal_) is then calculated as shown in Eq. (1):

1 $${\text{SA}}_{\text{metal}} = \left( {\frac{{V_{\text{ads}} }}{22,414}} \right) \cdot {\text{SF}} \cdot N_{\text{A}} \cdot A_{\text{metal}}$$

where SF is the stoichiometric factor of the number of adsorbed probe molecules per metallic surface atom, *A*_metal_ is the cross-sectional area of the metal and *N*_A_ is the Avogadro’s number.

The average metal particle size can then be calculated considering the geometry of the particle as shown in Eq. (2):

2 $${\text{SA}}_{\text{metal}} = \frac{A}{V \cdot \rho }$$

where *A* is the area of the particle, *V* is the volume of the particle and *ρ* is the density of the metal.

**Fig. 1:**
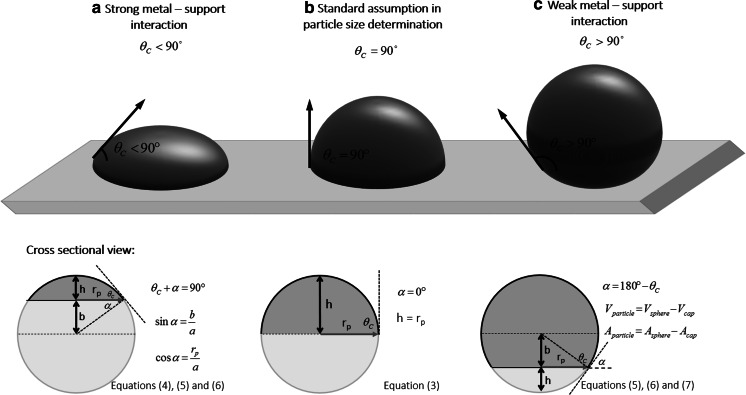
Effect of metal-support contact angle on the geometry of the metal nanoparticle

It is a common assumption that the metal nanoparticles consist of hemispherical clusters. As shown in Fig. 1b, the radius of the average metal particle (*r*_p_), based on Eq. (2), can be calculated with Eq. (3):

3 $${\text{SA}}_{\text{metal}} = \frac{A}{V \cdot \rho } = \frac{{2 \cdot \pi \cdot r_{\text{p}}^{2} }}{{\frac{2}{3} \cdot \pi \cdot r_{\text{p}}^{3} \cdot \rho }} = \frac{3}{{r_{\text{p}} \cdot \rho }}$$

However, currently a well-accepted fact in the field is that the metal deposition method affects not only the resulting particle size and its distribution but also the metal-support interaction (Haruta 1997). To give a few examples, the deposition–precipitation method usually yields to hemispherical metal particles where the flat planes of the metal attach to the support (Fig. 1b) while impregnation methods produce spherical particles simply loaded on the support (high contact angles, Fig. 1c) (Haruta 2002). Specifically in the case of gold nanoparticles, a certain metal-support interaction is essential for its catalytic activity, making the difference between a highly active and an inactive catalyst (Haruta et al. 1993). Additionally, we have recently demonstrated using high-angle annular dark field (HAADF) images taken in an aberration corrected STEM that when metal particles have a very strong interaction with the support, they present very small contact angles, with a morphology resembling plates (2D) rather than three dimensional particles (Torrente-Murciano et al. 2014, 2015) (Fig. 1a).

To illustrate the concept, let us consider the CO chemisorption analysis of platinum metal nanoparticles supported on an inert material with a metal loading of 1.5 wt%. For a given amount of chemisorbed carbon monoxide (*V*_ads_), the estimated average particle size can widely vary depending on the metal-support contact angle considered. For particles with strong metal-support interaction (*θ*_c_ < 90°), the average particle size can be estimated using Eq. (4) whose nomenclature is depicted in Fig. 1a.

4 $${\text{SA}}_{\text{metal}} = \frac{A}{V \cdot \rho } = \frac{{\pi \left( {r_{\text{p}}^{2} + h^{2} } \right)}}{{\frac{\pi \cdot h}{6}\left( {3r_{\text{p}}^{2} + h^{2} } \right) \cdot \rho }}$$

The particle height (*h*) can be calculated as

5 $$h = r_{\text{p}} - b = r_{\text{p}} \cdot \frac{{\left( {1 - \sin \alpha } \right)}}{\cos \alpha }$$

where *θ*_c_ is the contact angle between the metal particles and the support material and *b* is expressed as

6 $$b = \sin \alpha \cdot \frac{{r_{\text{p}} }}{\cos \alpha }$$

On the other hand, when the metal-support interaction is high (Fig. 1c), resulting in very small contact angles (*θ*_c_ > 90°), *b* and *h* are defined as before (Eqs. 5, 6) however, in this case

$$\propto = 180^{ \circ } - \theta_{\text{c}}$$

the average particle size can be calculated with Eq. (7):

7 $${\text{SA}}_{\text{metal}} = \frac{A}{V \cdot \rho } = \frac{{4 \cdot \pi \cdot \left( {b + h} \right)^{2} - \pi \left( {r_{\text{p}}^{2} + h^{2} } \right)}}{{\left[ {\frac{4}{3} \cdot \pi \cdot \left( {b + h} \right)^{3} - \frac{\pi \cdot h}{6}\left( {3r_{\text{p}}^{2} + h^{2} } \right)} \right] \cdot \rho }}$$

Metal dispersion (*D*) is the percentage of metal atoms accessible to the probe molecule, defined as

8 $$D = \frac{{V_{\text{ads}} \cdot {\text{SF}}}}{22,414} \cdot M_{\text{w}} \cdot 100$$

where *SF* is the stoichiometric factor and *M*_*w*_ the molecular weight of the metal/s.

As expected, for a given amount of CO adsorbed and a given contact angle, the average particle size increases when the metal dispersion decreases.

**Fig. 2:**
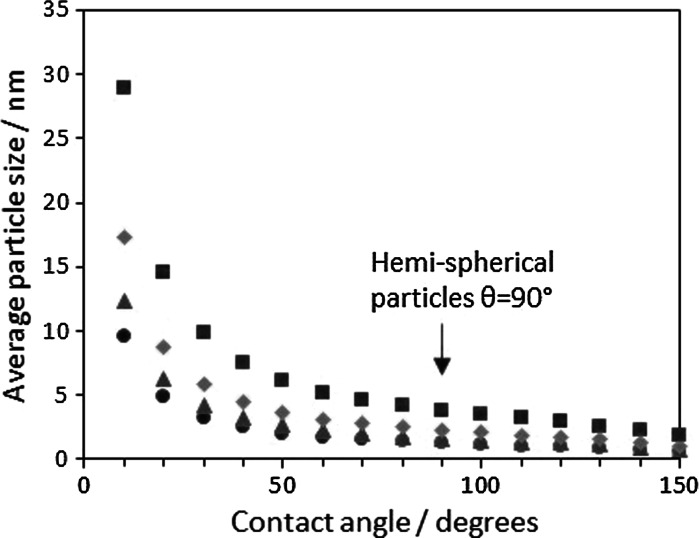
Effect of contact angle on the average particle size calculated by chemisorption for different metal dispersion values: *blue circle* 90 % *red triangle* 70 %, *green diamond* 50 %, *violet square* 30 %. (Color figure online)

**Fig. 3:**
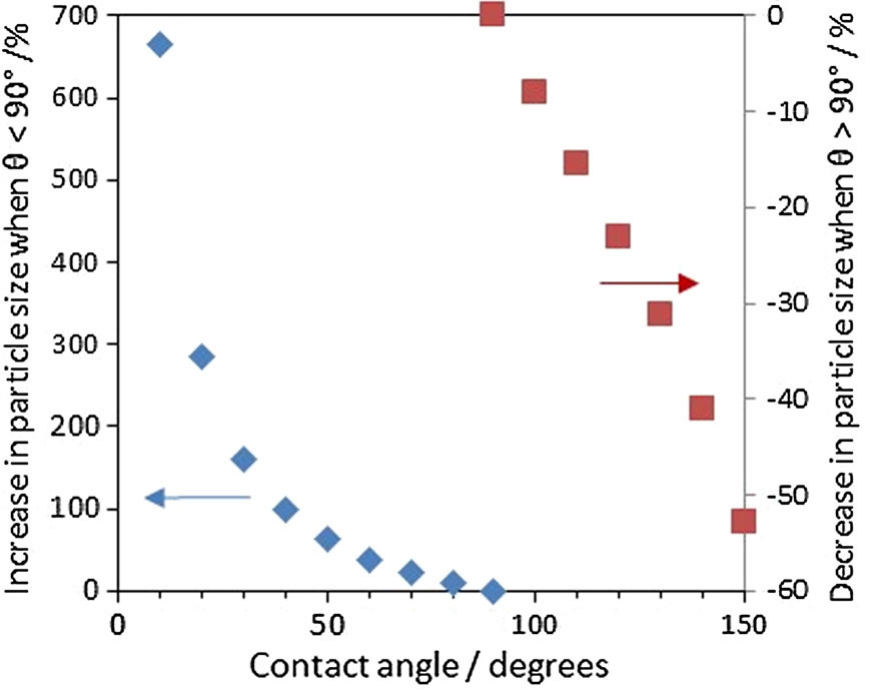
Percentage increase and decrease of particle size respect to the hemispherical shape (*θ* = 90°) when the contact angle is >90° (*blue* *diamond*) and <90° (*red square*), respectively. 70 % metal dispersion. (Color figure online)

The arrow in Fig. 2 indicates the estimated particle size assuming a hemispherical shape (*θ*_c_ = 90). When the metal-support interaction is weak and the contact angle >90°, the estimated particle size is slightly lower than that of the hemispherical shape. To give a few examples, the particle size is 23 and 52 % smaller when the contact angle is 120° and 150°, respectively, than that of a contact angle of 90° for a given amount of CO adsorbed (Fig. 3).

A more considerable effect is observed when there is a strong metal-support interaction resulting in contact angles <90°. The average particle size considerably increases as the contact angle decreases. Although the increase in particle size seems to be larger as the metal dispersion decreases (Fig. 2), the increase percentage is independent on the dispersion. The relationship between the actual particle size with respect to the one estimated considering a contact angle of 90° and the actual contact angle is shown Fig. 3. It is important to highlight that the conventional assumption of a hemispherical shape greatly underestimates the average particle size when there is a strong interaction between the metal and the support. To give a few examples, particle sizes are 100 and 660 % bigger than that of a hemispherical particle when the contact angles are 40° and 10°, respectively, for a given amount of CO adsorbed (Fig. 3).

The absolute variation on particle size (in percentage values) can be extrapolated to any metal or even bi-metallic systems, independently of their molecular weight, cross-sectional area and density. It is important to highlight that in the case of multiple metal systems, Eq. (1), and the values of metal cross-sectional area and density should be modified to consider such systems.

Another area of potential error in the determination of particle size by chemisorption is associated to the general assumption of spherical shapes which present the lowest surface area per volume ratio of all geometrical shapes. The literature contains numerous examples that illustrate the importance of the nanoparticle shape not only for catalytic applications but also in determining the chemical and physical properties of the particles (Li et al. 2008; Molina et al. 2011). It is important to note here that the potential particle size error determination by chemisorption is not related to the variations of adsorption strength of the probe molecules (such as carbon monoxide) to the different cluster facets (Yudanov et al. 2003). During chemisorption analyses, low temperature conditions, sometimes even requiring cryogenic conditions, ensure the saturation of the metal surfaces with the probe molecule, independently of geometry, facets and/or size (Guzman et al. 2009).

**Fig. 4:**
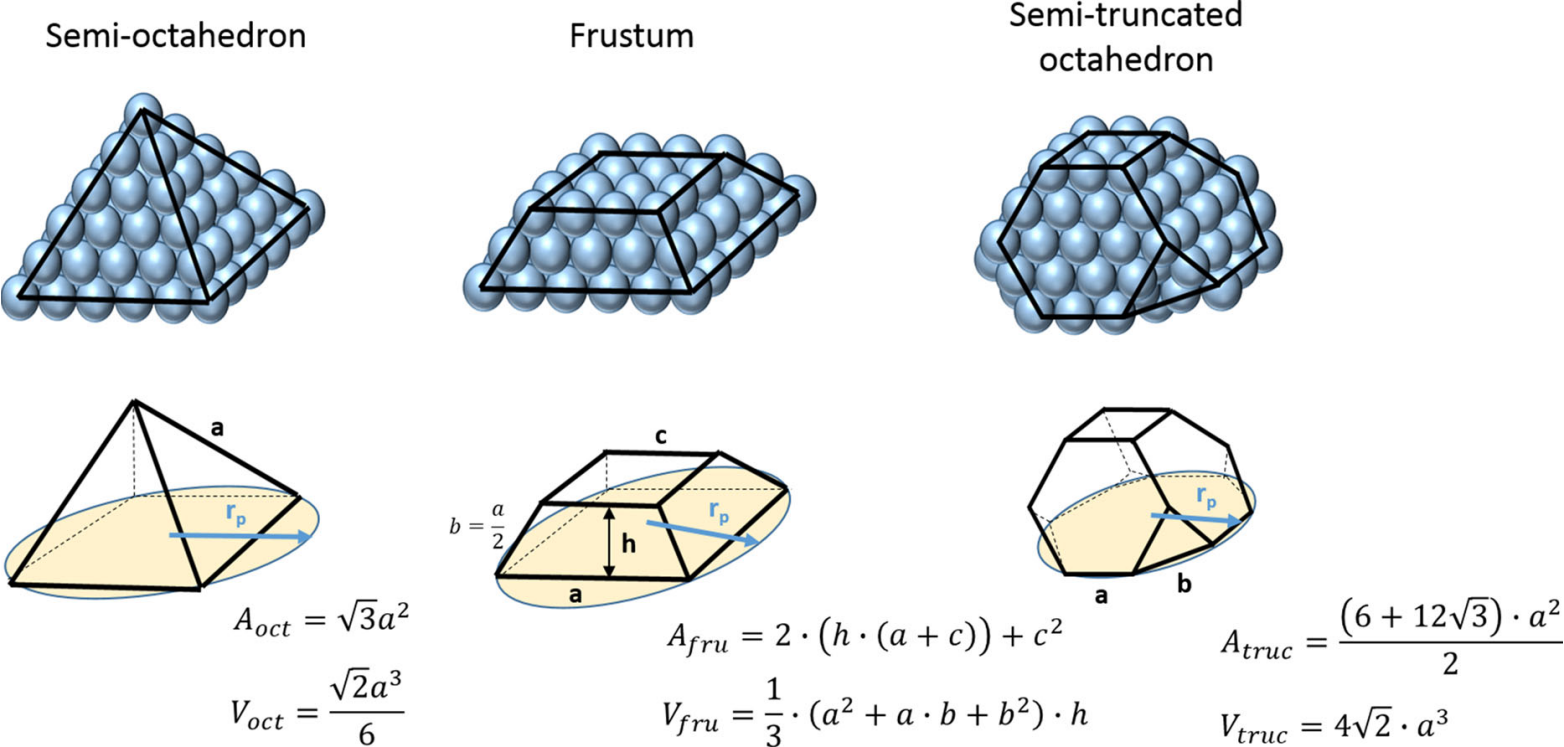
Effect of metal particle shape on the estimated particle size

To quantify the error associated to the assumption of spherical shapes in the metal nanoparticles in the estimated particle size, several 3D metal structures, widely observed experimentally for supported metal nanoparticles (Rossi et al. 2004; Yasumatsu and Fukui 2015), have been considering: semi-octahedron, semi-truncated octahedron and truncated pyramid (frustum), as shown in Fig. 4. The yellow circle represents the equivalent particle diameter of each morphology, which can be calculated taking into consideration the geometry of the particles as shown in the picture.

**Fig. 5:**
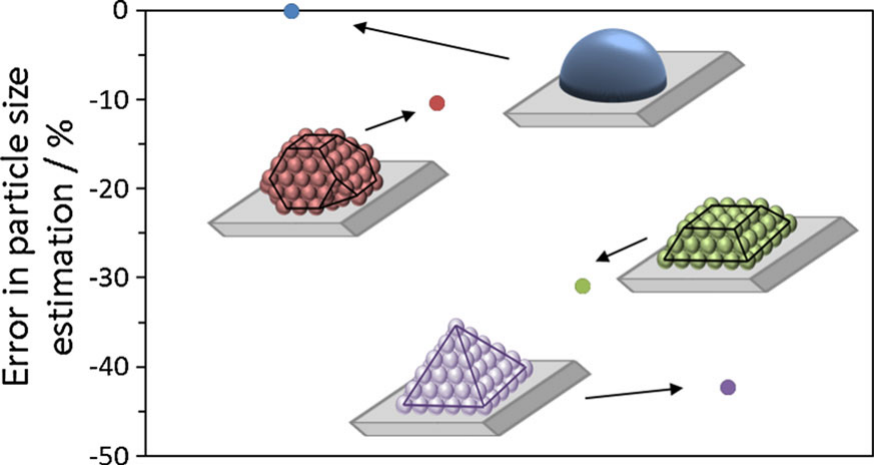
Error associated to the assumption of spherical shape (contact angle 90°) respect to other metal particle shapes

For a given amount of chemisorbed carbon monoxide (*V*_ads_), it is assumed that a semi-spherical particle shape, as conventionally done in chemisorption analysis, estimates the smallest average particle size compared to any other geometrical shape. Figure 5 shows that the particle size is slightly underestimated (~10 %) if a semi-spherical shape is assumed when the particle presents a semi-truncated octahedral shape. This error is considerably higher when the particles have more regular shapes such as semi-octahedron or a frustum, where it can account for ~30–50 %. The number of surface to bulk atoms, and consequently the amount of uncoordinated atoms, in these two particle shapes (semi-octahedron and frustum) is considerably higher than those in the semi-spherical shape particles, increasing their instability. Indeed, it has been demonstrated that the shape of the nanoparticles can instantaneously change into more thermodynamically stable forms (Hayakawa and Yasumatsu 2012).

It is important to highlight here that in all these cases of error estimation associated to the actual shape of the metal particles, the contact angle of the metal particles and the support is assumed to be 90°. The high error associated to the effect of the contact angle illustrated above for particles with spherical shapes is also applicable here. Moreover, stabilisation of well-defined metal facets and a high number of uncoordinated atoms normally requires a high metal-support interaction (Li et al. 2008) and in these cases, the error associated to this factor is considerably higher than that related to the actual particle shape.

**Fig. 6:**
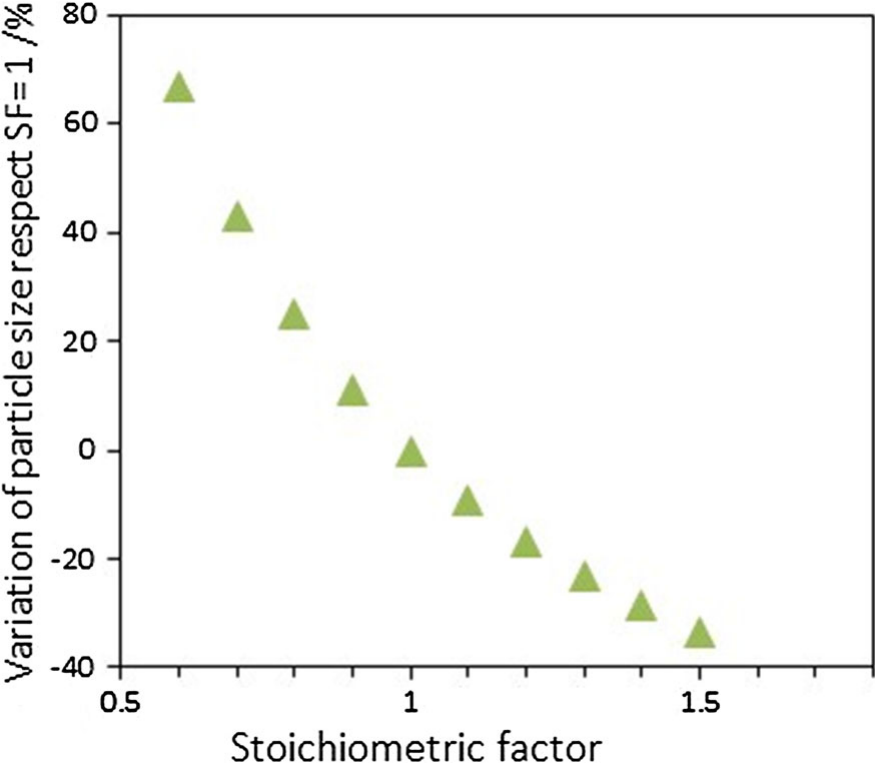
Percentage of particle size calculation as a function of the stoichiometric factor, taking SF = 1 as baseline

To put the misleading consideration of hemispherical particles during particle sizing using chemisorption into context, Fig. 6 shows the percentage error associated with an incorrect estimation of the stoichiometric factor as the number of probe molecules adsorbed per atom of metal, currently considered the main source of error in the estimation of particle size by chemisorption. Although the particle sizes can be considerably underestimated if the wrong SF is considered in the calculations, a significantly bigger error is associated with the lack of consideration of the metal-support contact angle, especially when there is a strong metal-support interaction (*θ* < 90°).

## Conclusions

The conventional assumption of metal particles with a semi-spherical shape for the calculation of average metal sizes using gas adsorption characterisation can provide misleading results if the metal particle does not have a hemispherical shape. Indeed, the metal-support interaction and consequently the resulting metal-support contact angle should be taken into consideration for accurate estimation of the average metal size. We have demonstrated that particle sizes are slightly overestimated when their contact angle is >90° (low interaction with the support); however, the particle sizes are greatly underestimated when their contact angle is <90° (high interaction with the support). In addition, we have also shown that this error is higher than that associated to the difference between the actual particle shape and the spherical assumption.
